# Impact of Relative Humidity on Wood Sample: A Climate Chamber Experimental Simulation Monitored by Digital Holographic Speckle Pattern Interferometry

**DOI:** 10.3390/jimaging5070065

**Published:** 2019-07-18

**Authors:** Vivi Tornari, Thomas Basset, Michalis Andrianakis, Kyriaki Kosma

**Affiliations:** 1 Foundation for Research and Technology-Hellas (FORTH), Institute of Electronic Structure and Laser (IESL), 100 N. Plastira str., Vassilika Vouton, 700 13 Heraklion, Crete, Greece; 2 Physics Department, Universite de Lyon, École Normale Supérieure de Lyon ENS, Université Claude Bernard Lyon I, 69100 Villeurbanne, France; 3 Institute of Plasma Physics and Lasers, Hellenic Mediterranean University, Tria Monastiria, 74100 Rethymnon, Crete, Greece

**Keywords:** wood, relative humidity, climate impact, deformation, sorption isotherm, holographic interferometry, digital holographic speckle pattern interferometry, DHSPI, cultural heritage

## Abstract

Relative humidity (RH) changes are a natural environmental effect that forces organic materials to a constant cycle of achieving equilibrium. The present work is part of an ongoing research based on the hypothesis that the inevitable deleterious effects of the RH natural cycle may be prevented or minimized if a deformation threshold is assigned to each monitored endangered object prior to exposure to structural damage. In this paper the characterization of the behavior of a softwood sample (1.0 cm thick) submitted to RH abrupt cycles has been performed, in terms of mass and rate of displacement of the surface. The exemplary study is based on the concept of recording the RH impact directly from the material surface, allowing us to identify diversity in reaction with time, which in turn could determine the onset of structural changes prior to irreversible damage. The RH impact is measured as surface deformation from interference fringes, using a custom-made real time holography system with interferometric precision termed digital holographic speckle-pattern interferometry (DHSPI). The main observations presented here are a hysteresis in the dynamic sorption isotherm and a greater rate of displacement during the drying. A long-term experiment was performed to identify signs of ageing of the sample. The evolution of the mass and the rate of displacement stayed similar, an offset with an interesting behavior was observed and highlights ageing of wood. In order to produce a future preventive model for distinct art objects it is necessary to determine a deformation threshold for each material. In this context the study was planned to continue with organic samples bearing variable density and thickness under longer-term RH cycles and monitoring until the samples show visible signs of irreversible damage.

## 1. Introduction

The impact of the environmental conditions and climate changes like the changes in relative humidity (RH) on organic art materials is worldwide studied as an essential preventive conservation measure in cultural heritage (CH) [1,2]. Artworks of organic composition, especially wood, are in constant interaction with the surrounding moisture in the environment [1,2,3]. The RH change effect to materials is a dimensional change, which steadily but invisibly causes irreversible structural damage: Depending on the ambient RH the material cycles of moisture adsorption and desorption is the dominant process for a material to maintain equilibrium with its environment; a process considered as most critical for physicochemical and structural alterations [4,5,6]. Organic materials based on carbon and hydrogen molecules are porous and characterized as hydrophilic. When the pores are of the order of thousands of a cm, as in the case of wood and cellulose, they have a large internal surface area allowing through capillary rise significant water absorption and moisture transmission. As water vapor molecules in the air are adsorbed to the internal surfaces the water content increases provoking structural expansion [7,8,9].

Wood plays a dominant role within materials used in cultural heritage (CH) since many art objects from paintings and icons to furniture and statues are made of wood, and/or art constructions are composed to a large extent of wood, therefore wood deterioration is a thoroughly studied subject [5,6,7,8,9,10,11,12,13,14,15,16]. In the last three decades concern for the safe maintenance of tangible and intangible CH has been accelerated due to fears for climate impact and phenomena closely related to climate change and/or extreme events that have tremendous consequence to CH preservation [17,18]. Climate change and pollution effects on CH have been studied through large European projects and it is widely accepted that climate impact affects the cycle phenomena in environmental alterations accelerating deterioration [19,20].

Laser holographic interferometry as a non-contact and non-destructive technique has been implemented to study remotely the materials’ reactions to environmental alterations in real time [21,22]. From the direct observation of materials’ reactions it has been concluded that materials react immediately to the RH change; hence, repeated surfaces displacements due to RH fluctuations as a consequence of climate impact would cause structural alterations that lead to material deterioration with irreversible structural damage and defect generation. The holographic interferometry technique has shown that the deformation is detectable and the deformation threshold exhibits variability, and as such can be classified by detecting the RH impact on organic materials according to different densities and thicknesses. Moreover, interferometry studies are performed to quantitatively measure the instantaneous response of materials to RH fluctuations, confirming that any reaction depends mostly on the condition of the object, the construction and material type; thus drawing a conclusion on quantitative differentiation from direct impact records on distinct materials and objects under simulation conditions is possible [23].

The “Noah’s Ark” European Commission research project [19] showed for the first time that climate change will have a severe impact on CH. The main climate changes threatening CH are the rising temperatures, enhanced amounts of precipitation and, as a consequence, a wider variability of RH, sea level rise and the shifting of the climatic zones. The “Noah’s Ark” project on climate change has among others concluded to set as the main research priority the study of the vulnerability of the typical CH materials through surface responses. In the next EC funded project “Climate for Culture” [20] the Noah’s Ark recommendation has been included into the project objectives. In this context, in the study of climate induced effects on movable and immovable CH pieces of art, a work-package related to the “experimental monitoring techniques for assessment of risk in the Climate Change era” was included, and the work-package was realized through: Surface inspecting technologies coupled with RH/temperature sensor readings, close-up 3D microscopy (3DM) determining surface damage phenomena, a new set of sensors being glass dosimeter (GD) providing synergetic environmental risk and the free-water-sensors (FWS) providing assessment of the available water content of the air together [24]. A nondestructive remote sensing system to record surface displacements as a response to the RH environment change was employed, making use of laser interferometry system termed digital holographic speckle pattern interferometry (DHSPI) [25]. The experimental results showed that mass and moisture content follow the RH change and produce subsequent surface and volume alterations [26,27].

Since the results of the non-contact, direct surface measurement technique were encouraging to the aim of further understanding the mechanisms of onset of fatigue, the experiments were further carried out towards the recording of the cycle of the absorption and adsorption mechanisms of wood, by studying the evolution of the equilibrium of moisture content as a function of the RH; in laboratory environment with constant temperature the analysis of the reaction in a sorption isotherm graph have been reported [14,28].

Hence in this paper we present further studies on the possibility of differentiation of displacement rates in relation to the surrounding environment as wood samples are forced by RH to follow the cycles of sorption isotherm and hysteresis, under constant climate-chamber temperature (T °C). The results prove that the DHSPI technique is a direct surface monitoring method and can be very useful towards the decisive prevention strategies on collections and monuments. 

## 2. Materials and Methods

### 2.1. Experimental Aim

The global aim of the direct monitoring of materials in simulated cycles of environmental conditions is the study of the natural ageing process that could highlight our knowledge on how and when irreversible damages finally appear. The presented study was part of the ongoing research on this topic performed in combination with other techniques and models [23,24,26,27]. In this study in particular investigation aiming on highlighting wood sample reactions to temporal variations of the RH was described. In case of wood under RH fluctuations and moisture content (MC) values the thermodynamic equilibrium was not reached, but rather non-equilibrium thermodynamics was involved. A lot of questions remain for the phenomena at equilibrium not to mention at non-equilibrium. The fundamental element was to understand the temporal evolution of wood when the RH changes. In usual outdoor or indoor conditions wood is exposed to periodic humidity changes, daily, seasonal and annual. Research on the reaction of wood samples to these changes is scarce, but several experimental studies report on some very interesting general results [10,12,13,14].

- For periodic fluctuations of the RH (period of several hours), equilibrium is not reached.
- There can be a phase lag between the temporal evolutions of the RH and of the MC.
- The MC and the RH show different rates of temporal evolution due to the hysteresis between the adsorption and the desorption and the non-linear relation between the MC and the RH.

A theoretical simulation can be a difficult task due to the complex structure of wood. The simplest and most simplified model is a moisture diffusion model, which uses the Fick’s law with cyclic boundary conditions. This model gives good results [27] when applied to experimental data [26] showing that abrupt short-term fluctuations of the RH could be of great danger for wood. Thus here a study on the impact of cyclic abrupt short-term fluctuations of the RH on wood (around 40% RH in some hours) was presented.

#### 2.1.1. Experimental Methodology: Climate Chamber-DHSPI Workstation and Measurement Method

**Figure 1:**
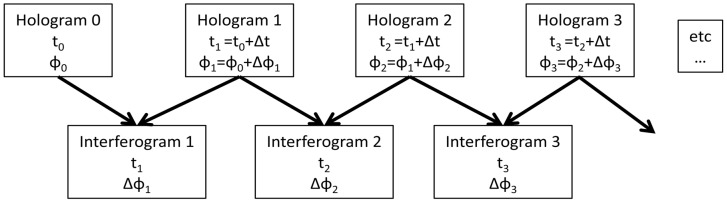
Scheme of interferogram generation from data collected from the climate chamber-Digital Holographic Speckle-Pattern Interferometry (DHSPI) workstation.

In order to measure micrometric surface displacements on a wood sample due to fluctuations of the RH, the DHSPI system that is based on the holographic interferometry technology was employed [25,26,27]. Holography is a method to create a fully three-dimensional image of an object, which is called a hologram. Contrary to a classical photography, which is a two-dimensional image formed by a lens, this method enables us to record the full wavefront scattered by an object, and so its three-dimensional image. Thus a hologram records the phase and the amplitude of the incident field, whereas classical photography is just sensitive to the intensity of light. Digital holographic speckle-pattern interferometry (DHSPI), is based on off-axis digital holographic speckle pattern interferometry. Speckle-pattern refers to a complex interference pattern generated by multiple interferences. The term speckle is usually used to describe the granular aspect of a laser beam on an optically rough surface (height variation of the order of or greater than the wavelength), due to multiple interferences between the light scattered by each point of the surface. Digital refers to a numerical technique used to record and read a hologram. Holographic interferometry refers to a technique that enables us to measure the displacement of the optically rough surface of an object with an interferometric precision (fractions of the wavelength) by overlapping holograms [28,29,30,31,32]. The principle of this technique is summed up in the scheme of Figure 1. 

A hologram was recorded every given time interval Δt and the phase *φ*_i_ of each hologram was extracted; the subtraction of two consecutive phases gives a phase difference Δ*φ*_i_, which is directly linked with the displacement occurred during Δt. The resulting interferograms represent the phase difference within the specified time interval. It is noted that the sequence of interferograms are not generated by the subtraction of each record from the initial one as in classical double exposure interferometry [29], but from the immediately precedent one [33]. The recording of data took place in the climate chamber while the RH was increased or decreased to provoke the reactions of the materials. This methodology allows for relative displacements within specific time intervals and hence the estimation of "rate of change" of material within specific environmental change. The methodology is very useful in dynamic effect visualization and suitable for environmental impact assessment. 

#### 2.1.2. Optical Geometry

**Figure 2:**
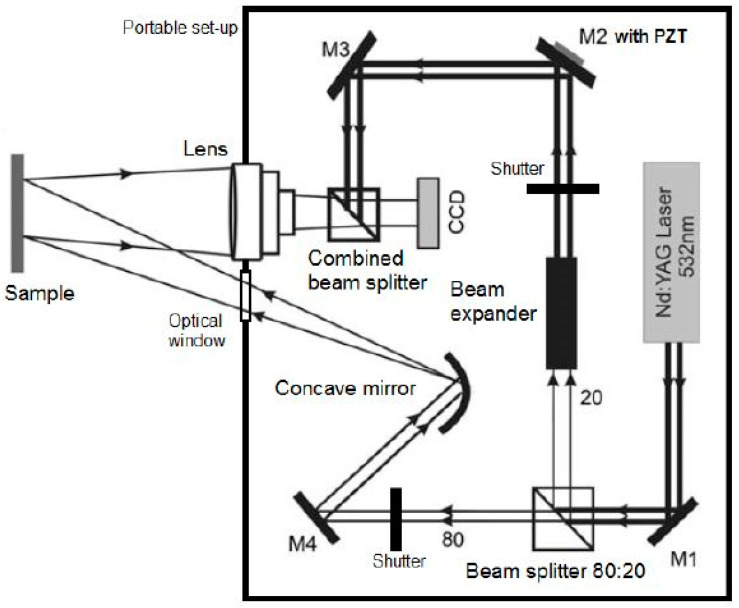
The optical geometry of the DHSPI system follows two paths that are driven by optical elements to produce an interference between the object and an unmodulated beam into a CCD camera.

The optical geometry of the DHSPI system is designed according to the strict boundary conditions for optical off-axis transmission holography in double exposure phase-shifted holographic interferometry mode of application (DE psHI). The geometry of the portable instrument is shown in Figure 2 and development is described in detail elsewhere [21,22,34].

The variable beam splitter (shown as cube beam splitter) divides the collimated laser beam in two beams, which follow different optical paths. The beam with 20% of laser intensity is the reference beam, which is directed to the CCD camera through the mirrors M2–M3, while the rest of the beam (object beam) illuminates the object/sample through the path driven by mirror M4 and the Concave mirror-expander. The light scattered from the object surface re-enters the system to interfere with the reference beam, through a combining beam splitter, onto the CCD. It is noted that the two beams interfere via the combined beam splitter before the CCD to generate an image plane hologram. The characteristics of the CCD camera (IDS) are: 5 MP resolution and 3.45 μm pixel size. To generate the required phase-shifted interferograms the angle of the reference mirror M2 is driven modulo 2π by a piezo-electric transducer (PZT). For each interferogram five phase steps images are recorded as *I*_1_ to *I*_5_ speckle holographic intensities at one time-interval (Δ*t*_1_) and five phase steps recorded at *I*_1_ to *I*_5_ speckle holographic intensities at another time-interval (Δ*t*_2_) are recorded, in order to deduce the phase difference Δ*φ*_2-1_ [35,36], with the phase shifting algorithm set as:

(1) $$\tan\left(\varphi \right)=\frac{2\left(I_{2}-\;I_{4}\right)}{2I_{3}-I_{5}-I_{1}}.$$

#### 2.1.3. Measurement of Surface Optical Displacement

**Figure 3:**
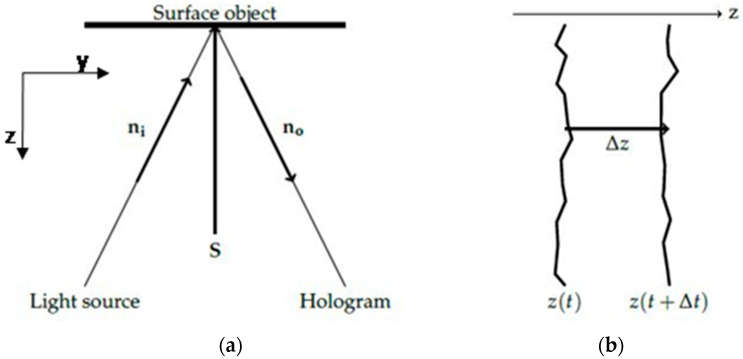
(**a**) Surface illumination configuration and observation, and (**b**) main surface displacement direction.

In holographic interferometry an expanded laser beam illuminates the surface of the investigated object. The surface rests in one position and then an excitation is applied to provoke a change in surface spatial coordinates [34]. Thus the surface subjected to a load is displaced to another position as shown in the scheme of Figure 3: In the case of dimensionally responsive materials the dominant displacement is perpendicular to the surface, thus along the z-axis (Figure 3a,b), while x- and y-axis changes are considered negligible. This means that we captured and measured the out of plane component of deformation, in which the DHSPI system based on the off-axis holographic interferometry was mostly sensitive.

In holographic interferometry, we define the sensitivity vector *S* as:

(2) $$S=\frac{2\pi }{\lambda }\left(n_{i}-n_{0}\right),$$

with *n_i_* the surface illumination direction and *n*_0_ the observation direction. The surface displacement induces a phase difference Δ*φ* between two successive holograms.

(3) Δφ=S d,

with S *d*, the displacement vector of the surface [29,36].

In out-of-plane recording, as in the present scheme, the orthogonal illumination is *n_i_* = −*n*_0_ and the phase difference is directly linked to the displacement along the vector normal to the surface.

(4) $$\Delta \varphi =\frac{4\pi }{\lambda }\Delta z,$$

with Δ*z* the displacement along the *z* axis (Figure 3).

This phase is represented in the interferogram fringe pattern

(5) $$I=I_{0}\left[1+\cos\left(\Delta \varphi \right)\right].$$

The number of fringes *N* of the fringe pattern is directly linked to the phase difference Δ*φ*.

(6) $$N=\frac{\Delta \varphi }{2\pi }.$$

Thus the displacement Δ*z* is directly linked to number of fringes *N*.

(7) Δz=Nλ/2.

The measurement allows us to capture shrinking and swelling of wood due to environmental alterations to RH. Since the wood surface as a reaction to RH is just a few μm the technique that measures in multiples of half wavelength provides the highest possible sensitivity to visualize and study the reaction of the material. Imperceptible internal changes in wood structure can be detected witnessing the procedure of the aging of wood. In this context, the phase difference within specific time intervals indicates the rate of change of the surface and hence is linked to the rate of displacement. To determine the rate of surface deformation as a response to environmental fluctuations within a period of time the rate of displacement (RoD) is deduced from the direct measurement of the number of fringes N of each interferogram:

(8) $$RoD=\frac{\Delta z}{\Delta t}=\frac{N\lambda /2}{\Delta t}.$$

In the present experiments the time interval between the recording of two holograms was set at Δ*t* = 3 min, the laser wavelength was with *λ* = 532 nm and Δ*z* the displacement along the z-axis as in Figure 3, while fringe measurement was performed from 0 order fringe along the axis of fringe distribution. Further details on the method with which the number of fringes *N* was determined from an interferogram can be found in [29,32,34].

### 2.2. Workstation Geometry, Salts and Sample

**Figure 4:**
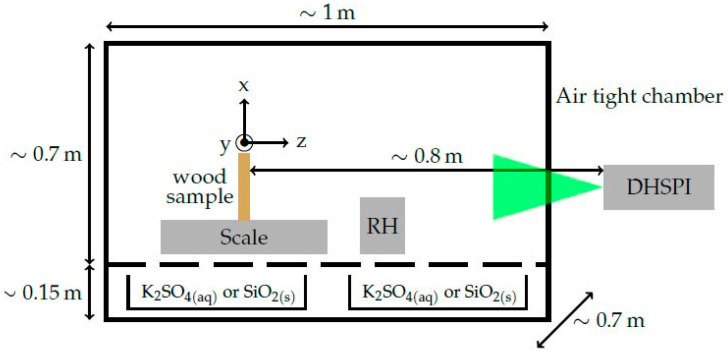
Schematic of the airtight climate chamber-DHSPI monitoring workstation.

The DHSPI system was set to face the surface of the free-standing sample, which was located inside a climate chamber carrying salt-solution cases, environmental sensors and airtight construction. The scheme of the workstation in Figure 4 shows the airtight climate chamber in the on-line workstation along with the DHSPI system. The wood sample was mounted on a digital scale, the RH/T data loggers were placed on the side and the salt solutions on a special case underneath, allowing for changing the conditions inside the chamber.

**Figure 5:**
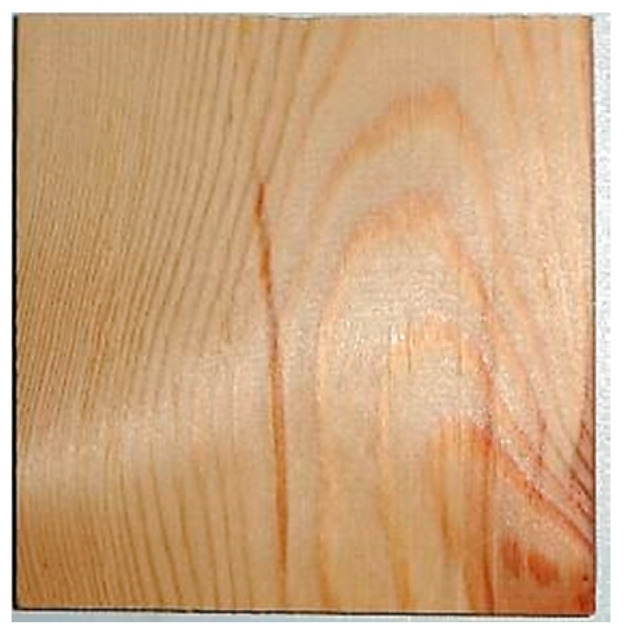
Photo of softwood sample pine tangential cut.

**Figure 6:**
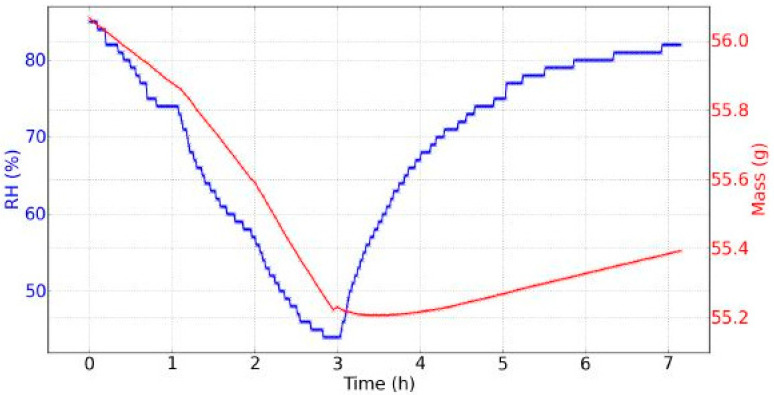
Evolution of the relative humidity (RH) and mass during seven hours, day 7 of experiments.

The combination of saturated potassium sulfate salt solution and silica gel resulted in the decrease of the RH from 85% to 44% in three hours, and a following increase from 44% to 82% in another four hours. The sample (Figure 5) was a 1-cm thick softwood pine, tangential-cut piece, 10 cm × 10 cm in size, with material density ~0.552 to ~0.56 g/cm^3^. Considering that the volume of the sample remained stable over the period of seven hours (10 cm × 10 cm × 1 cm) and according to the mass data of Figure 6 (variation between ~55.2 g and ~56 g), the density of the sample varied from ~0.552 to ~0.56 g/cm^3^.

The RH change was used to provoke the transient effect on wood in order to observe its responses. Wood is a hygroscopic material that can exchange by sorption (absorption and adsorption) vapor water with the surrounding air until it reaches the equilibrium moisture content. This MC was determined by the environment. A first idea is to consider that the water density in the air determines the MC. Although the latter indicates the absolute humidity, it was not relevant in this study. Indeed with the same absolute humidity a wood sample in hot air is dry and in cold air is damp [1]. Therefore a more relevant quantity is the relative humidity (RH), the ratio between the water vapor pressure and the saturating water vapor pressure, that is, RH% = P_vap_/P_sat_ (T) × 100%. P_sat_(T) increases with temperature, and therefore wood in hot air is drier due to the weaker relative humidity, compared to colder air. Thus the RH is the relevant scale and the MC increases with RH. The main effect of the moisture content variations is the dimensional changes of wood. A wood sample swells when its MC increases, i.e., the RH increases, and vice versa. 

A typical graph showing the interaction in the evolution of the equilibrium moisture content as a function of the relative humidity at a constant temperature is called sorption isotherm. In a typical sorption isotherm graph [14] two isotherms are shown, due to a first drying from green wood to dry wood or first cycle, and then a clear hysteresis between the desorption and adsorption isotherms or second cycle. The hysteresis is always observed with wood. For different woods, the mean ratio between the two curves is around 0.8 [14]. The maximum MC is nearly the fiber saturation point (FSP) that is at 99% RH. To understand these isotherms one should first understand the phenomena taking place in wood. Water exists in three forms in wood: Capillary water, vapor and hygroscopically bound water, but only bound water explains the typical sigmoid isotherm graph. Since below the FSP vapor water in the lumen has a negligible influence and dry wood is not a porous material under conditions below nearly 99.5% RH, there is no capillary sorption [37,38]. Thus, these isotherms are mainly influenced by the adsorption of water on hydroxyl groups on the surface of the cell wall composed of hemicellulose, cellulose and lignin via hydrogen bonding. Actually the typical sigmoid shape is a type II sorption isotherm in the BET theory of adsorption of a gas by a solid surface (IUPAC 1985 classification) [14]. However, there is no physically consistent model, which explains all the experimental observations, especially the hysteresis.

Hysteresis is common with capillary phenomena, but the capillary sorption is negligible. This hysteresis is linked with adsorption with no definitive explanation. A qualitative explanation is that during drying, water is removed and the hydroxyl groups, which were bounded with water are not satisfied anymore, so they move close together to form hydrogen bonds. Then, during rehydration, less hydroxyl groups are available so the moisture content increases “slowly” [14,39].

### 2.3. Experimental Procedure

The sample was set for several days in stable RH/T conditions inside the climate chamber before change in RH condition was applied. What was being monitored was:

(1) Real time RH and T, monitoring and logging;
(2) Real time mass recordings of the sample, and;
(3) Surface displacement, monitored with DHSPI.

In this experiment the evolution of three parameters was studied:

- The RH/T was measured in real-time with a hygrometer (accuracy ≈ 3%) and temperature logger placed inside the air-tight chamber (temperature resolution of 0.4 °C). To control the RH inside the chamber saturated salt solutions of potassium sulfate (high RH) and silica gel as desiccant (low RH) were employed.
- The mass of the wood sample (placed in a free standing position and taped on the scale) was measured in real-time with a precision of 1 mg.
- The surface displacement of the sample along the z-axis (orthogonal to the surface) was monitored in real-time by DHSPI. The DHSPI portable device was on the axis with the sample outside the chamber and illuminated its full surface with an expanded laser beam.
- With respect to the laboratory temperature outside the climate chamber during the experiments, the laboratory temperature was kept relatively constant with smooth fluctuations from a minimum of 24 °C during some nights to a maximum during some days of 28 °C. These variations were gradual with very low rate of change.

After the change in the salt solution the recording of the sample reactions started, as shown in Figure 1, while readings from the hygrometer, the temperature and weight were simultaneously acquired and electronically archived in specific software environments.

## 3. Results

The saturated salt solution of potassium sulfate alternated with silica gel provided a cycle of 85% RH to 44% RH in three hours, then a 82% RH in four hours was reached. During drying, the evolution was nearly linear. During rehydration, the evolution followed an exponential rising curve, becoming more and more slow. In Figure 6 the evolution of the RH and the mass of the wood sample on the 7th day of the experiments is shown. The amplitude of variation of the mass was 0.86 g. It was observed that during the drying out process the mass "follows" the RH, i.e., their temporal evolutions were similar, while during rehydration, the mass evolved slower with an almost linear evolution of 0.057 g/h rate change (last three hours). Moreover, a “rebound” in the evolution of mass was observed when the RH began to increase: Mass reached its minimum after the RH had reached its minimum. This time lag was equal to: Δt = tMmin − tRHmin = 34 min.

**Figure 7:**
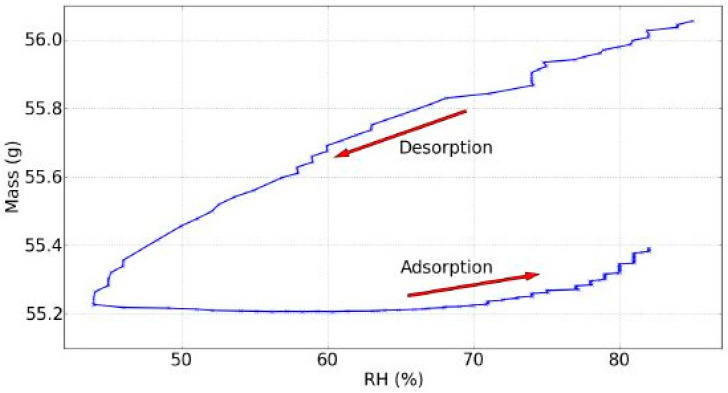
Dynamic sorption isotherm, day 7 of experiments.

With the available data, the dynamic sorption isotherm at non-equilibrium could be plotted and is shown in Figure 7. A hysteresis was witnessed due to the mass that did not follow the same RH rate change during the rehydration, but rather increased with another characteristic shape linked with this specific evolution of the RH.

**Figure 8:**
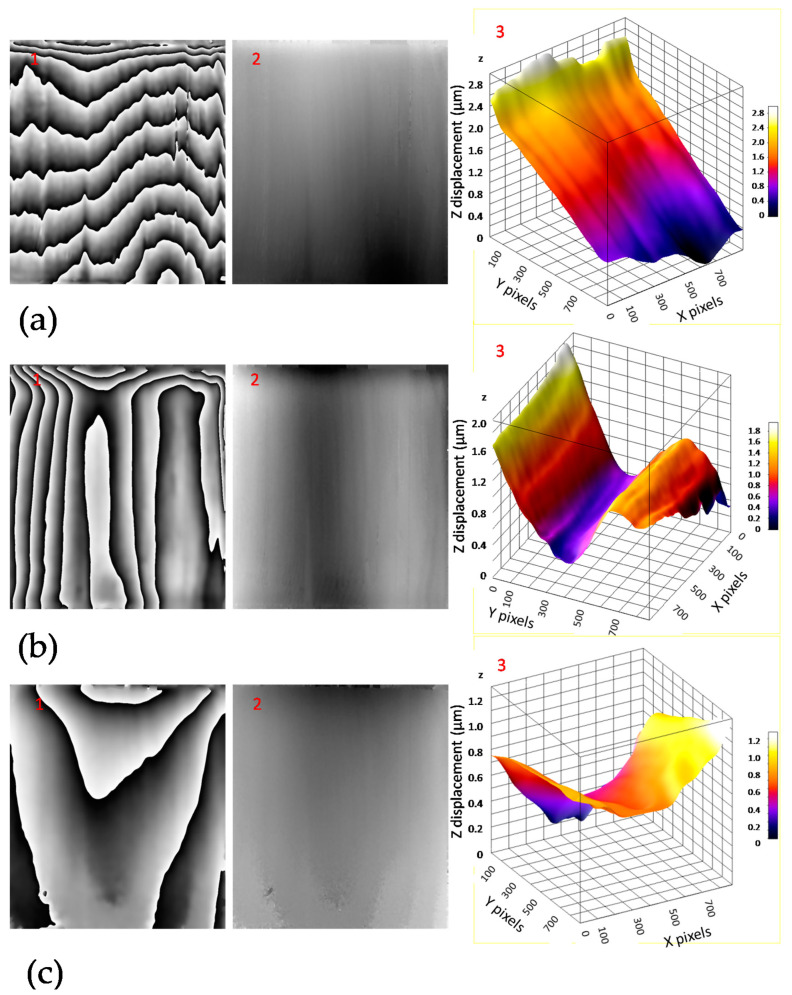
Examples of filtered interferograms (1), the unwrapped-phase (2) and three-dimensional surface deformation maps (3), data related to Figure 6, at (**a**) 12 minutes after the beginning of the cycle (drying), (**b**) 231 minutes from the beginning of the cycle (rehydration start) and (**c**) 416 minutes from the beginning of the cycle (mass characteristic plateau). (In plot 8b3 the x,y axes are rotated in order to show better the surface displacement).

**Figure 9:**
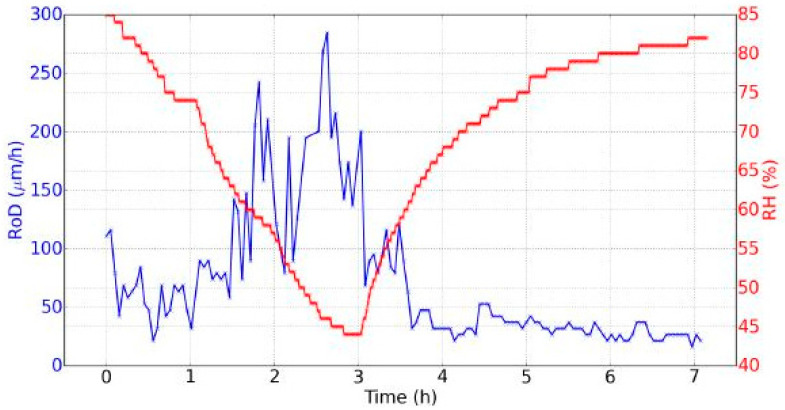
Evolution of the RH and the rate of displacement (RoD) during seven hours (day 7, one point every minute for the RH, and one point every three minutes for the RoD).

Figure 8a–c shows an a indicative sequence of interferograms obtained within seven hours of measurements during one experimental day, at 12, 231, and 416 minutes after the beginning of the cycle. The interferogram (wrapped phase) is shown in (1), the unwrapped phase in (2) and the 3D surface graph in (3). Such interferograms were obtained with the DHSPI system for all the twenty days the experiment was running, and every three minutes during the seven hours of experiment for each day. The processing (wrapped–unwrapped phase) and post-processing (measurement of surface displacement, 3D graph visualization) of the recorded sequential interferograms in full field mode of operation (the whole surface simultaneously) provided all the information regarding the surface displacement data related to this climate chamber experimental work. The measurement was performed with the special 5-phase shifted algorithm software [22,25] and processing of the captured differential images provided the number of fringes (N) and the surface displacement (Δφ), allowing for the calculation of the rate of displacement (RoD) as described in the previous sections. Figure 9 shows the RoD during day 7 [23]. 

We could observe two main points regarding the temporal evolution of the RoD (Figure 9).

**Figure 10:**
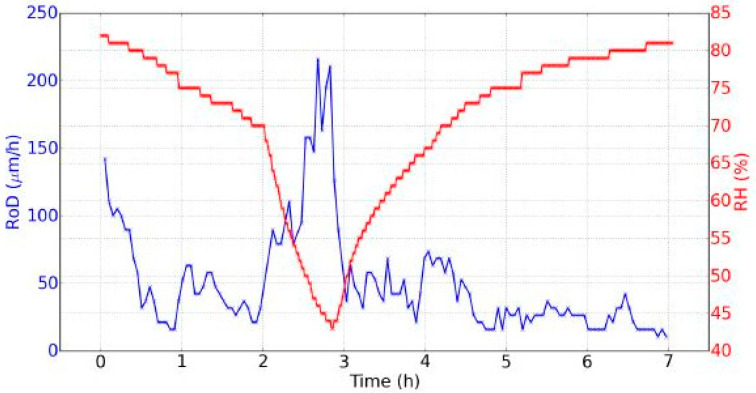
Evolution of the RH and the RoD during seven hours (day 2, one point every minute for the RH, and one point every three minutes for the RoD).

- The RoD was clearly greater during drying. This means that there was a great difference between the two phenomena of drying and rehydration, linked with the hysteresis of the dynamic sorption isotherm (Figure 7).
- During drying, the RoD was greater after one hour and a half when the RH decreased faster. At the beginning of the rehydration, the RoD was more significant during the first thirty minutes, and then fell as the RH evolved more and more slow. Therefore the RoD seemed to be correlated with the temporal derivative of the RH. The faster the RH changed, the higher the RoD was. This effect was better demonstrated on the data from day 2 shown in Figure 10, where two different temporal evolutions of the RH during drying can be noted.

**Figure 11:**
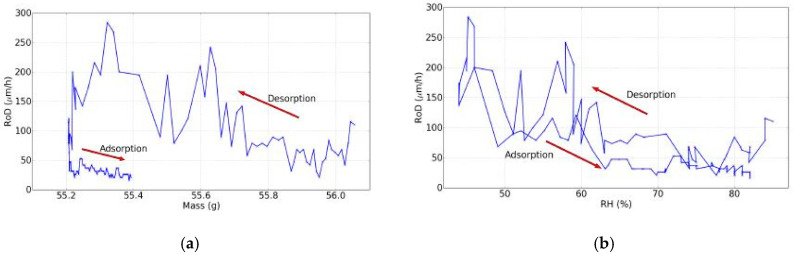
(**a**) The RoD as a function of the mass and (**b**) the RoD as a function of the RH (day 7).

Next, the RoD was plotted as a function of mass shown in Figure 11a and of the RH is shown in Figure 10b, in the same way as the dynamic sorption isotherm (Figure 7). These curves were characterized by hysteresis too.

**Figure 12:**
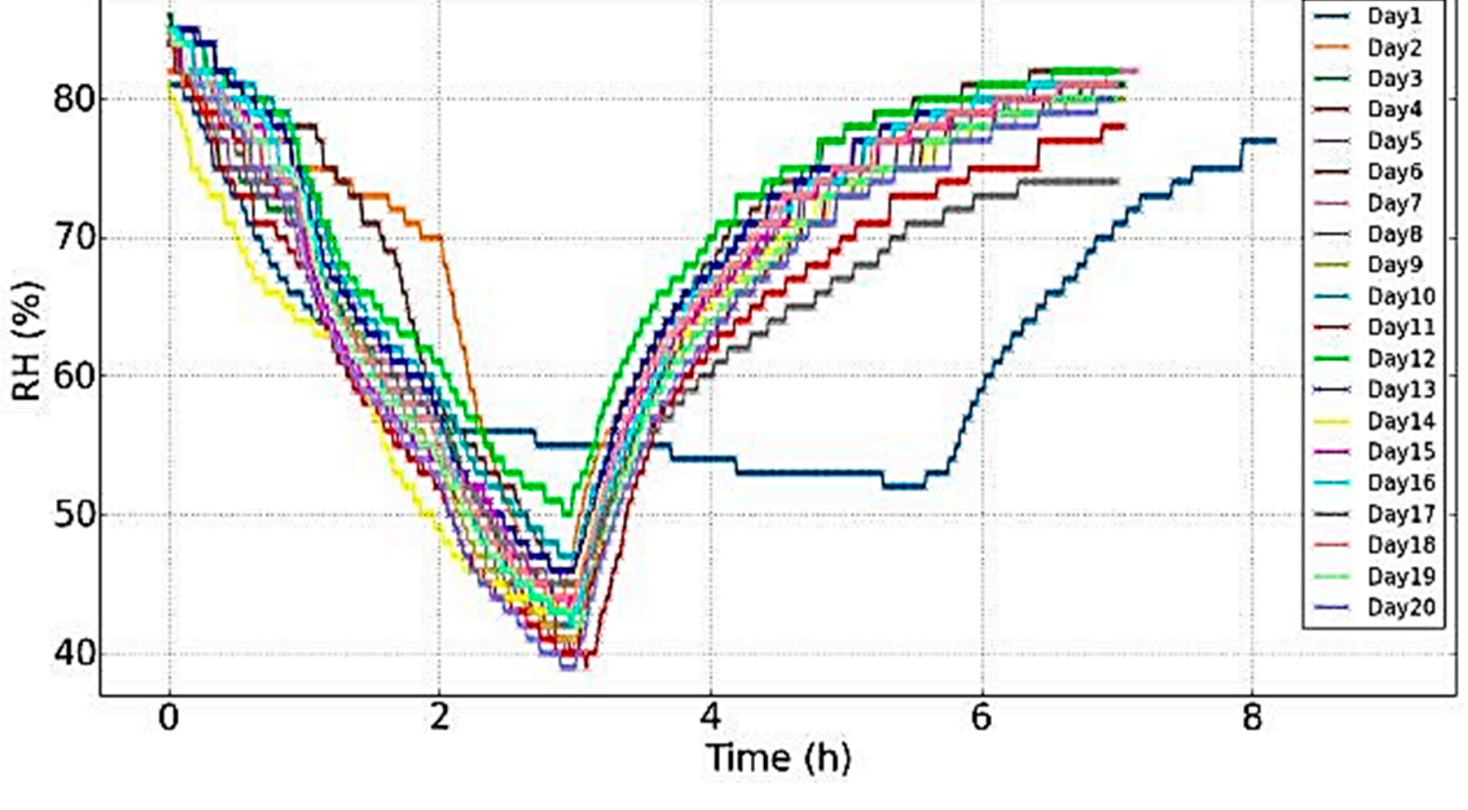
Evolution of the RH during the twenty days of experiments.

The same RH cycle was repeated during an evolution of twenty days (Figure 12) and was similar for each day of the experiments: A fall of nearly 40% during the first three hours, a rise during the next four hours and during night to reach the previous initial value were observed. Although the RH change was introduced with saturated salts solutions in the same manner variations in the RH evolution could arise (e.g., day 1) due to parameters that could not be fully controlled, like the interaction of the RH in the chamber with the materials’ hysteresis and the different moisture content during different days of measurements (especially day 1), affecting the absorbance capacity. 

Figure 12 shows the recordings of the mass following the evolution of RH in consecutive days. The evolutions were similar with the mass always following the RH during the drying out process, while during re-hydration of the climate chamber the re-absorption of moisture was a slowed down process with slower temporal evolution. There was only an offset between the curves. 

**Figure 13:**
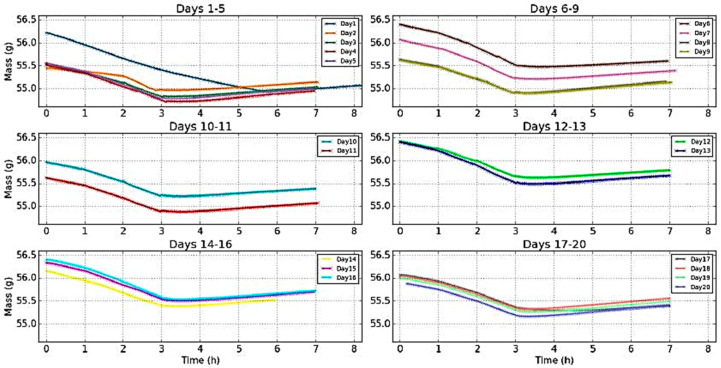
Evolution of the mass during the twenty days of experiments.

From the graphs of Figure 13, where the cycle shown in Figure 12 was repeated for all 20 days with settling periods of relaxation in between these days, it was observed:

1. Days 1–5: After day 1, the sample could not reach its previous initial mass. The curves for days 1–4 were "blocked" in a zone below 55.5 g and were superimposed.
2. Days 6–9: After relaxation of three days, the sample had time to reach its initial mass of the day 1 (a little bit higher). Then there was a descending offset and the curves of days 8 and 9 were superimposed. This zone where the curves were "blocked" was a little bit higher than the corresponding zone for the days 1–5 (difference around 0.1 g).
3. Days 10–11: After relaxation for two days, the evolution was similar to the previous period.
4. Days 12–13: After relaxation for fifteen days, the curves were "blocked" in a higher zone above 55.5 g.
5. Days 14–16: After relaxation for eleven days, the curves were "blocked" in the same zone.
6. Days 17–20: After relaxation for two days, the offset decreased and the curves were "blocked" in a lower zone with a minimum value around 55.2 g.

**Figure 14:**
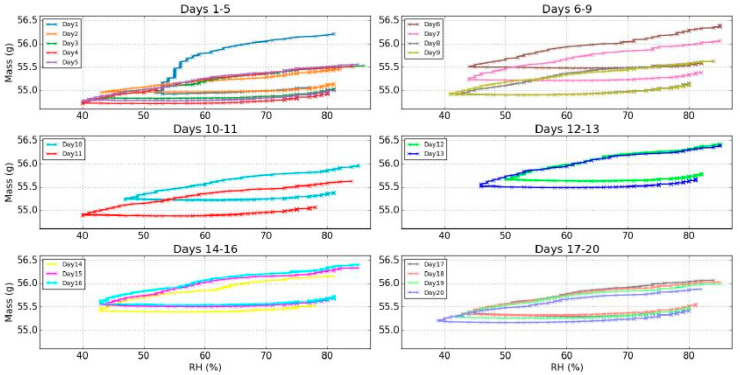
Evolution of the isotherms during the twenty days of experiments.

Thus, even though the evolutions were similar, the wood sample presented signs of internal changes. Indeed, for the changes in the minimum mass this value increased (days 1–16), and then decreased (days 17–20) without reaching the lowest minimum mass (day 4 with 54.7 g). It is noticeable that the evolution of the mass for day 17 was really slow during the rehydration (delayed minimum and smaller slope). This was due to an unusual slower evolution of the RH. To erase these more or less significant variations of the RH for each experiment, the plot of the dynamic sorption isotherms, which shows the same evolution of the offset was performed (Figure 14).

**Figure 15:**
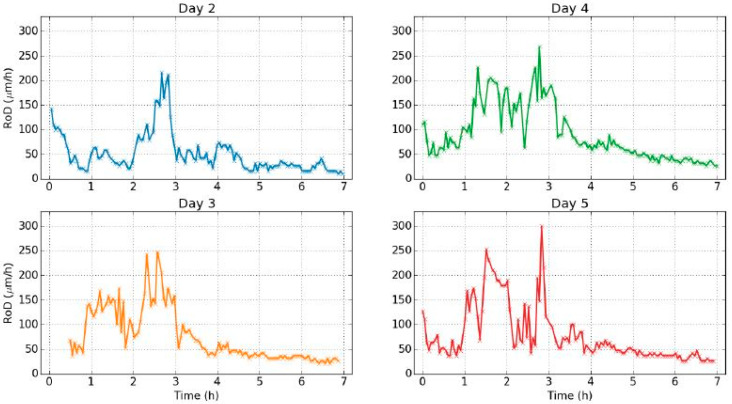

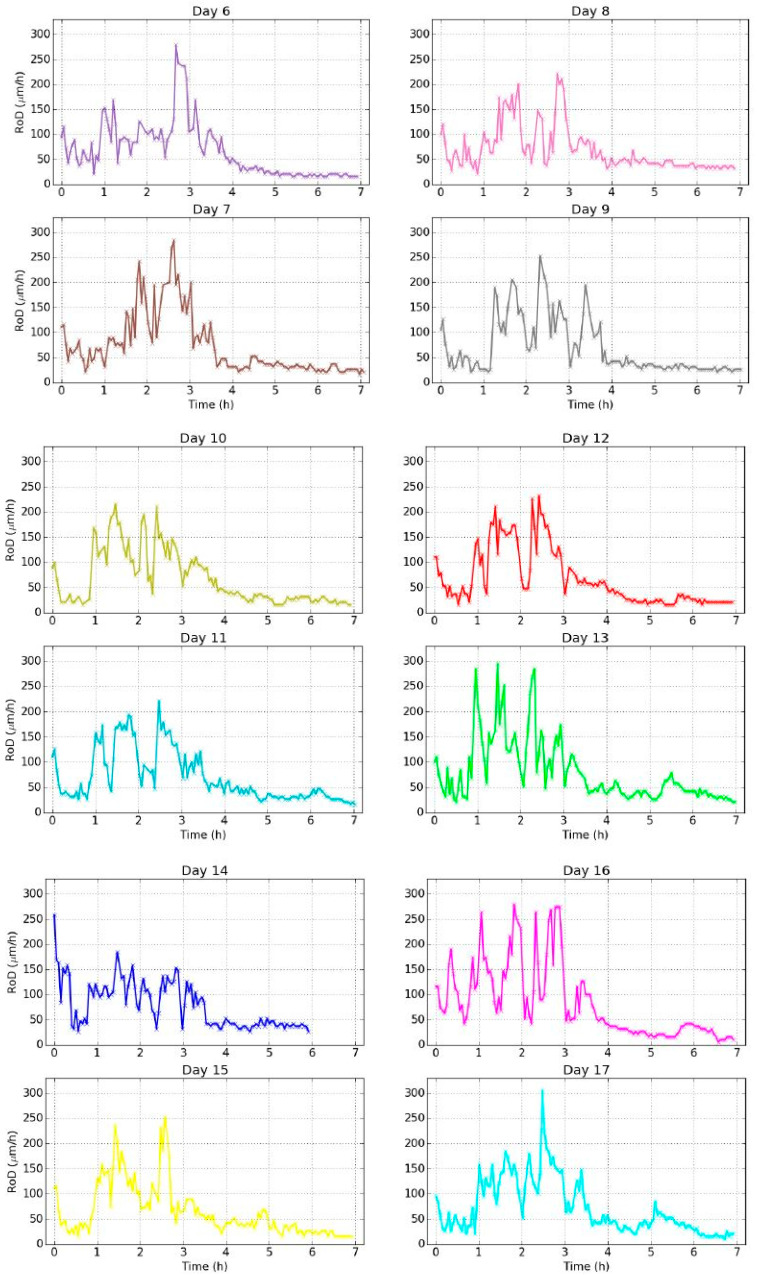

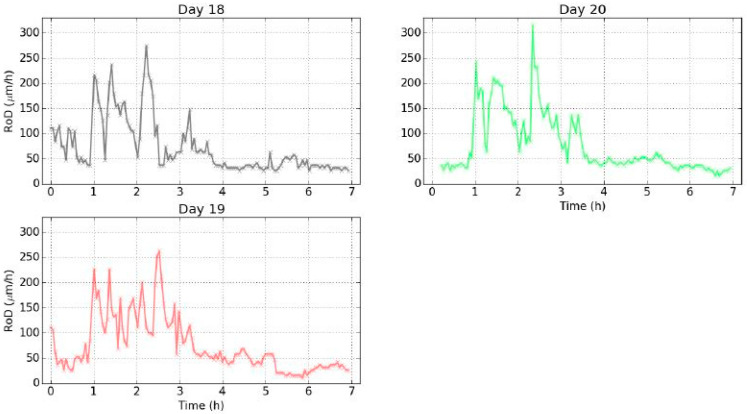
Evolution of RoD from the 2nd to 19th day of experiment.

It should be noted here that the shape of the isotherm plot from day 1 was differentiated from the rest curves. This reaction was rather an expected one: In environmental change experiments and especially in controlled climate chamber experiments, values commonly exhibit on the first day a rather different reaction before stabilization is achieved in the rest of the days. An obvious answer is the inertia of material due to the shock of change from a previous equilibrium, to altered conditions. After a period of days the material exhibited a hysteresis loop, hence it was like it recognized and adjusted to the change in a stable and repeated manner. As such the RoD of day 1 was not shown since reaction value was established in day 2 as the graphs of Figure 15 represent.

The studied evolution of the RoD is shown in Figure 15 in chronological order.

During the total of twenty days of experiments, similar evolutions with the same characteristics were obtained. It is possible that twenty days were not enough to observe variations. The greatest problem was the fact that the experimental days involved relaxation times for the wood sample to achieve initial mass. This is a probable reason that greater internal changes in the sample could not be developed. As a next step in the experiment, longer experimental cycles within the tested values without relaxation time for the sample should be set. 

## 4. Conclusions

The first aim of this study was to characterize the behavior of the wood sample submitted to RH cycles. Concerning the mass was observed that it adapted fast to the RH during the drying process, a phenomenon directly linked with the well-known hysteresis of the sorption isotherm, which was observed in the dynamic sorption isotherm. The measurements with DHSPI have enabled us to measure the rate of displacement of the surface due to the variation of the RH. A high RoD was observed during the drying and a lower RoD during the rehydration, an observation that illustrates again the difference between the drying and the rehydration process. Moreover the RoD seemed to be correlated with the temporal rate of evolution of the RH. The long-term experiments showed that, during several days, it observed the same reaction and described a behavior pattern of the material. Past experiments have showed that this statement can be claimed for many different types of materials and complex samples. The DHSPI system was confirmed to be an effective recording system for monitoring reactions to environmental impact directly from the examined object.

The evolution of the mass presented a clear offset, whose evolution seemed to be linked with ageing of wood. No significant variation in the evolution of the RoD was observed. A future experiment should be designed such as not to allow periods of sample relaxation but rather keep constant RH cycles during consecutive days. Actually the relaxation periods within the experiments did not allow us to study the ageing of wood under conditions where, in the real case scenario, the RH fluctuations, within a number of different values, is the everyday reality. It was designed to also submit the wood sample to faster RH cycles hence with smaller time windows between the minimum–maximum values in order to simulate more and faster RH variations in case of extreme climate events.

With respect to the monitoring system it was confirmed that DHSPI enabled us to measure the displacement of the surface of a wood sample when it swelled and shrinked due to variations of the RH. This technique is highly relevant to this study for many important reasons. DHSPI allows for high accuracy of the order of half-wavelength (*λ* = 532 nm), with the displacements of the wood surface being of the order of some μm. It thus signifies that imperceptible external or internal changes can be detected (non visible to the naked eye and to other known techniques employed in conservation or other fields) due to ageing of wood. Moreover, this technique records automatically in pre-specified intervals in a non-contact, non-invasive, non-destructive and full-field manner, all of which offer great advantages to in-situ measurements of materials deformations and to the study of artworks and historical buildings in response to their environmental surroundings, since they are based on an optical technique that does not have shape or surface roughness limitations. The DHSPI technique also enables us to measure real-time displacement and to do a full-field surface monitoring of complex 3D objects. It is very important that DHSPI records out-of-plane displacement that is more relevant component in dimensionally responsive materials escaping from measurement by other techniques. Last but not least the rate of deformation (RoD) provides a value that can be classified to provide an early warning signal of deformation for preventive conservation strategies in cultural heritage protection.
